# Adrenergic receptors: a key determinant of outcomes in bacterial pneumonia and bacterial sepsis-associated acute lung injury/acute respiratory distress syndrome through participation in pulmonary innate immune response

**DOI:** 10.3389/fimmu.2026.1744464

**Published:** 2026-07-09

**Authors:** Lin Zeng, Jinshu Yang, Jiangtian Yan

**Affiliations:** 1 LiShizhen College of Traditional Chinese Medicine, Huanggang Normal University, Huanggang, Hubei, China; 2 Hubei Key Laboratory of Germplasm Improvement and Utilization of Dabie Shan Dao-di Herbs (Huanggang Normal University), Huanggang, Hubei, China; 3 LiShizhen Culture and Industry Research Center of Traditional Chinese Medicine, Huanggang, Hubei, China

**Keywords:** acute lung injury, acute respiratory distress syndrome, adrenergic receptors, inflammation, innate immune responses

## Abstract

Acute lung injury (ALI) and its most severe form, acute respiratory distress syndrome (ARDS), are life-threatening inflammatory syndromes driven by dysregulated pulmonary innate immune responses, with bacterial pneumonia and non-pulmonary sepsis representing the leading etiologies. Despite decades of extensive preclinical and clinical research, no disease-modifying pharmacological therapies have been established for ALI/ARDS, with all-cause mortality remaining persistently between 40% and 60%. The onset and progression of ALI/ARDS are universally accompanied by robust release of the endogenous catecholamines epinephrine (EPI) and norepinephrine (NE), which signal via adrenergic receptors (ARs) expressed on nearly all structural and immune cell populations in the lung. ARs are G protein-coupled receptors (GPCRs) that serve as the core molecular interface between the sympathetic nervous system (SNS) and pulmonary innate immunity, modulating inflammatory responses, barrier integrity, and pathogen clearance within the lung. Recent seminal studies have delineated central neural circuits governing lung inflammation via sympathetic–adrenergic signaling, defined cell subset-specific bidirectional functions of ARs activation, and yielded critical mechanistic insights into the longstanding adrenergic paradox, substantially advancing our neuroimmunological understanding of ALI/ARDS pathogenesis. In this review, we systematically delineate the cell-type-specific and disease context-dependent roles of ARs subtypes in regulating pulmonary innate immune responses in both direct (pneumonia-induced) and indirect (sepsis-induced) ALI/ARDS. We critically integrate the canonical and non-canonical signaling pathways of ARs, and address the longstanding “adrenergic paradox”—the context-dependent bidirectional effects of ARs signaling, which can both suppress excessive inflammation and impair host antibacterial defense. We further examine the translational challenges of ARs-targeted therapies, including the failure of prior clinical trials, and provide a mechanistic framework for the development of optimized therapeutic strategies for ALI/ARDS.

## 1 Introduction

Acute respiratory distress syndrome (ARDS) is an acute, diffuse inflammatory lung injury defined by the 2012 Berlin Consensus criteria, characterized by increased pulmonary vascular permeability, elevated lung weight, and loss of aerated lung tissue (1). First described in 1967 (2), ARDS constitutes the most severe end of the disease continuum, while acute lung injury (ALI) is used to describe the full spectrum of this inflammatory syndrome (3). ALI/ARDS is driven by unrestrained cytokine storms and the breakdown of the alveolar-capillary barrier, with hallmark pathological alterations including inflammatory cascade-mediated damage to alveolar epithelial cells (AECs) and vascular endothelial cells (ECs), diffuse pulmonary interstitial and alveolar edema, and progressive respiratory failure (4–8). Epidemiological studies estimate a crude incidence rate of ALI of 78.9 per 100,000 person-years, with an age-adjusted incidence of 86.2 per 100,000 person-years (9). The majority of clinical studies report all-cause mortality rates ranging from 40% to 60%, with both incidence and mortality rising progressively with advancing age (10). Despite substantial advances in supportive care—most notably lung-protective mechanical ventilation—no disease-modifying, efficacious pharmacological therapies for ALI/ARDS have been established to date (11). This critical gap underscores an urgent, unmet clinical need to identify druggable regulators of the dysregulated inflammatory responses that drive ALI/ARDS progression.

Pneumonia and non-pulmonary sepsis are the primary etiologies of ALI/ARDS, which are predominantly associated with bacterial infections encompassing both Gram-negative and Gram-positive species (11–13). Notably, ARDS onset—whether triggered by direct pulmonary infection with *Escherichia coli* or systemic inflammation induced by intravenous bacterial toxins—is universally accompanied by robust release of endogenous epinephrine (EPI) and norepinephrine (NE) into the systemic circulation (14, 15). EPI synthesis and release occur primarily within the adrenal medulla, whereas NE is produced at sympathetic nerve terminals in both the peripheral nervous system and the brain (16). It is well established that both catecholamines signal via binding to adrenergic receptors (ARs)—a family of G protein-coupled receptors (GPCRs) broadly expressed throughout the central nervous system and nearly all peripheral tissues, including innate immune cells and non-professional immune cells within the lung (16–18). Under both physiological and pathological conditions, the release of EPI and NE, and their subsequent activation of ARs on innate immune cells, constitute the core pathway mediating crosstalk between the brain and the immune system, regulating both protective and pathological inflammatory responses (19). Extensive research has demonstrated that excessive release of catecholamines drives ARDS pathophysiology via ARs activation (20, 21), and that AR signaling-modulated innate immune responses are critical determinants of both infection resolution and the progression of inflammatory tissue damage in the lung (22, 23).

Although ARs are well-established as core mediators of sympathetic–immune crosstalk within the pulmonary compartment, seminal recent studies have delineated two parallel brain–lung neural circuits that govern ALI/ARDS progression: a protective circuit centered on hypothalamic corticotropin-releasing hormone (CRH) neurons, sympathetic efferent nerves, and neutrophil β2-AR signaling, and a pro-inflammatory circuit mediated by central amygdala (CeA) γ-aminobutyric acid (GABA)ergic neurons, sympathetic nerves, and β2-AR signaling in interstitial macrophages (IMs). These findings advance our mechanistic understanding of the hierarchical central regulatory network upstream of pulmonary AR signaling, and provide a unifying mechanistic framework for the context-dependent bidirectional effects of adrenergic signaling in pulmonary inflammation (21, 24).

While prior studies have delineated the expression and functional roles of ARs in the lung, the field currently lacks a unified, critical framework that integrates the cell-type-specific effects of AR subtypes across the two dominant etiologies of ALI/ARDS. Furthermore, there remains a paucity of systematic analyses addressing the translational barriers associated with therapeutic targeting of this pathway. In this review, we systematically delineate the multifaceted roles of ARs in modulating pulmonary innate immune responses in both bacterial pneumonia- and sepsis-associated ALI/ARDS. We further critically interrogate the context-dependent bidirectional effects of AR signaling, integrate the core signaling axes that drive cell-type-specific functional outcomes, and dissect the key barriers to clinical translation of ARs-targeted therapies. The overarching goal of this review is to establish a rigorous mechanistic foundation to guide future basic research and rational therapeutic development for ALI/ARDS.

## 2 Pathogenesis of ALI/ARDS and the pulmonary innate immune landscape

The pathogenesis of ALI/ARDS, irrespective of inciting etiology, is universally driven by dysregulated, unrestrained innate immune responses within the pulmonary compartment (25). As a primary mucosal immune organ, the lung contains a highly integrated cellular network of professional innate immune cells, including macrophages, innate lymphoid cells (ILCs), dendritic cells (DCs), and neutrophils, alongside non-professional immune cells such as airway and alveolar epithelial cells and vascular ECs—all of which express pattern recognition receptors (PRRs) that detect microbial and endogenous danger signals (26, 27). These PRRs include transmembrane Toll-like receptors (TLRs) and C-type lectin receptors (CLRs), in addition to cytoplasmic NOD-like receptors (NLRs) and RIG-I-like receptors (RLRs) (27), all of which recognize conserved pathogen-associated molecular patterns (PAMPs) including lipopolysaccharide (LPS) from Gram-negative bacteria, peptidoglycan (PGN) from Gram-positive bacteria, bacterial flagellins, and microbial nucleic acids (28). Innate immune cells also detect damage-associated molecular patterns (DAMPs) released by injured or dying cells in the setting of inflammation, including high mobility group box protein 1 (HMGB1), extracellular ATP, mitochondrial DNA, and S100 family proteins (29, 30). Detection of PAMPs and DAMPs via PRRs triggers transcriptional upregulation of inflammatory genes, thereby driving neutrophil recruitment into the alveolar space, progressive injury to the alveolar-capillary membrane, and impaired tissue repair—all of which represent the defining pathological hallmarks of ALI/ARDS (31, 32).

**Figure 1:**
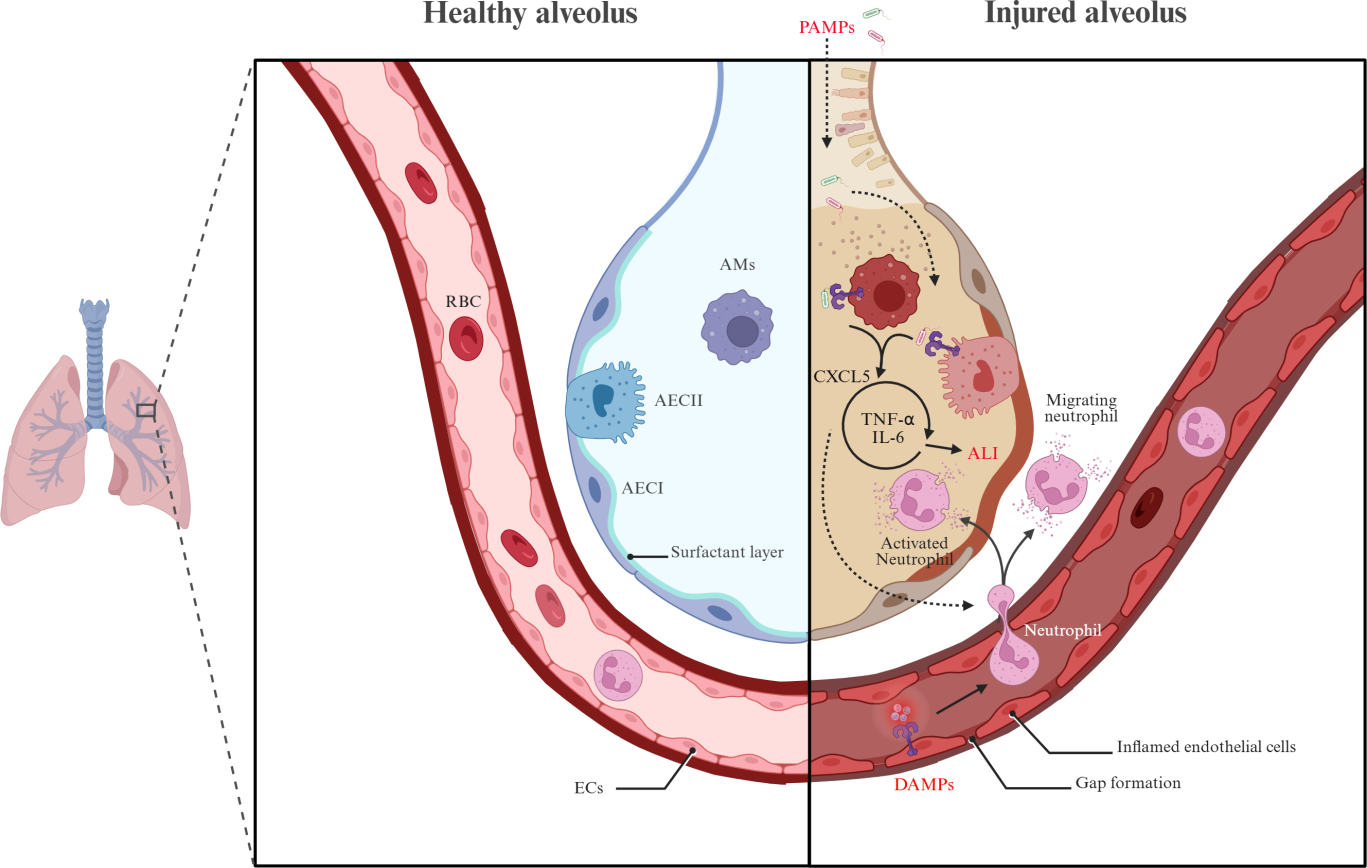
Pulmonary innate immune response during bacterial pneumonia/sepsis-associated ALI/ARDS. AECI, alveolar type I epithelial cell, AECII, alveolar type II epithelial cell; ALI, acute lung injury; AMs, alveolar macrophages; ECs, endothelial cells; TLR, Toll-like receptor.

Critically, the two dominant etiologies of ALI/ARDS drive disease pathogenesis through distinct primary injury pathways, which differentially shape the pulmonary innate immune response and the functional outcomes of AR signaling. Bacterial pneumonia, the most common cause of direct lung injury, is broadly categorized into two clinical entities: community-acquired pneumonia (CAP), most frequently attributed to *Streptococcus pneumoniae*, and hospital-acquired pneumonia (HAP), most commonly caused by *Staphylococcus aureus*, *Pseudomonas aeruginosa*, *Klebsiella* spp., *Escherichia coli*, *Acinetobacter* spp., and *Enterobacter* spp (12). Upon invasion of the lower respiratory tract by pathogenic bacteria, innate immune and structural cells within the alveoli detect bacterial PAMPs via PRRs. This recognition triggers the secretion of pro-inflammatory cytokines, including tumor necrosis factor-α (TNF-α), interleukin (IL)-1β, IL-6, and IL-8, alongside chemokines such as CXC chemokine ligand 5 (CXCL5) (22). This process initiates a cascade of local inflammatory responses and neutrophil chemotaxis, a host response evolved to eradicate invading pathogens. However, excessive, unrestrained inflammation can inflict damage on adjacent healthy pulmonary epithelial cells, leading to severe pneumonia-associated ALI/ARDS (22) (**Figure 1**).

In contrast, sepsis—which confers the highest risk of progression to ALI/ARDS and accounts for approximately 40% of all clinical cases—causes indirect lung injury driven by systemic inflammation (10). Sepsis is formally defined as life-threatening organ dysfunction resulting from a dysregulated host response to infection, most commonly arising from Gram-positive or Gram-negative bacterial infections, with the incidence of viral sepsis rising substantially during pandemic periods (13, 33, 34). In the setting of sepsis-induced indirect ALI/ARDS, PRRs expressed on pulmonary vascular ECs detect circulating PAMPs and DAMPs (22). This recognition drives ECs activation, vascular hyperpermeability, and the transmigration of circulating neutrophils and monocytes into the alveolar space (22). This inflammatory cascade initiates and amplifies pulmonary inflammation, ultimately culminating in severe ALI/ARDS (22) (**Figure 1**). Whereas both etiologies converge on dysregulated pulmonary innate immunity as a final common pathway, the primary cellular targets of injury differ fundamentally: pneumonia-induced direct ALI/ARDS is defined by primary damage to the airway and alveolar epithelial barrier, whereas sepsis-induced indirect ALI/ARDS is characterized by primary disruption of the pulmonary vascular endothelial barrier, with distinct ARs-mediated regulatory effects in each disease context.

## 3 Adrenergic receptors signaling: canonical pathways and the context-dependent adrenergic paradox

ARs are a class of GPCRs defined by their canonical structure, comprising seven hydrophobic transmembrane domains, an intracellular C-terminal domain, an extracellular N-terminal domain, and three paired intracellular and extracellular loops (35). Based on sequence homology and signaling properties, these receptors are categorized into three primary subfamilies: α1 (α1A, α1B, α1D), α2 (α2A, α2B, α2C), and β (β1, β2, β3) receptors. These receptor subfamilies are broadly expressed across the central nervous system and nearly all peripheral tissues, including all key structural and immune cell populations within the pulmonary compartment (17, 18). This paper provides a synopsis of ARs-related G proteins, the distribution of ARs, and relevant therapeutic drugs, drawing upon extant research in the field (19, 35, 36) (**Table 1**). The endogenous agonists of ARs are NE and EPI, which exhibit distinct potency hierarchies across receptor subfamilies: NE displays higher potency than EPI at α1- and α2-ARs, whereas EPI is more potent than NE at β-ARs (19). Adrenergic catecholamines (EPI and NE) released into the systemic circulation during physiological or pathological stress bind to ARs to regulate core homeostatic functions (37). Sympathetic nerves innervating the adrenal medulla mediate EPI and NE synthesis and release into the systemic circulation via the sympathetic–adrenal–medullary (SAM) axis (38). In addition, the vagal-adrenal axis has been shown to modulate adrenal catecholamine release (39). Moreover, activation of the sympathetic nervous system (SNS)—a ubiquitous network of sympathetic nerves that innervate effector cells across virtually all organ systems—drives local NE release from presynaptic nerve terminals (40). Locally released NE subsequently binds to α and β AR subtypes on adjacent effector cells via a paracrine mode of action, thereby mediating rapid cellular responses to stress (40).

**Table 1:** Adrenergic receptor-associated G proteins, distribution of adrenergic receptors, and associated therapeutic drugs.

Receptor types	Associated G proteins	Main transduction mechanisms	Tissues	Immune cells	Therapeutic drugs (indications)
α1A	G_q_/11(G_q)_	phospholipase C stimulation, calcium channel	Cerebral cortex, cerebellum, heart, liver, prostate, urethra	Lymphocytes, macrophages, monocytes, dendritic cells, neutrophils, NK cells	Agonists: methoxamine, methylnoradrenaline, midodrine, oxymetazoline, metaraminol, phenylephrine (vasoconstriction and mydriasis, used as vasopressors, nasal decongestants, and eye exams) Antagonists: alfuzosin, doxazosin, phenoxybenzamine, phentolamine, prazosin, tamsulosin, terazosin, trazodone (hypertension, benign prostatic hyperplasia)
α1B			Spleen, kidney, somatic arteries and veins, heart, endothelial cells, osteoblasts	Lymphocytes, macrophages, monocytes, dendritic cells, neutrophils, NK cells	
α1D			Cerebral cortex, heart, aorta, blood vessels of prostate, bladder	Lymphocytes, macrophages, monocytes, dendritic cells, neutrophils, NK cells	
α2A	G_i_/G_o_	adenylate cyclase inhibition, potassium channel, calcium channel, phospholipase A2 stimulation	Brain, spleen, kidney, aorta, lung, skeletal muscle, heart, liver	Lymphocytes, macrophages, monocytes, dendritic cells, neutrophils, NK cells	Agonists: dexmedetomidine, medetomidine, romifidine, clonidine, brimonidine, detomidine, lofexidine, xylazine, tizanidine, guanfacine, amitraz (antihypertensives, sedatives and treatment of opiate dependence and alcohol withdrawal symptoms) Antagonists: phentolamine, yohimbine, idazoxan, atipamezole, trazodone, mianserin, mirtazapine (aphrodisiac, antidepressants, reversal of α2-ARs agonist-induced sedation)
α2B			Kidney, liver, brain, lung, heart, cardiac muscle, skeletal muscle, aorta, spleen	Lymphocytes, macrophages, monocytes, dendritic cells, neutrophils, NK cells	
α2C			Brain, kidney, spleen, aorta, heart, liver, lung, skeletal muscle	Lymphocytes, macrophages, monocytes, dendritic cells, neutrophils, NK cells	
β1	G_s_	adenylate cyclase stimulation	Brain, lung, spleen, heart, kidney, liver, muscle	Lymphocytes, macrophages, monocytes, dendritic cells, neutrophils	Agonists: Dobutamine, isoprenaline, noradrenaline (bradycardia, heart failure, cardiogenic shock) Antagonists: Metoprolol, atenolol, bisoprolol, propranolol, timolol, nebivolol (cardiac arrhythmia, congestive heart failure, glaucoma, myocardial infarction, migraine prophylaxis)
β2			Brain, lung, skin, liver, heart	Lymphocytes, macrophages, monocytes, dendritic cells, neutrophils, NK cells	Agonists: (short-acting) salbutamol (albuterol), levosalbutamol (levalbuterol), terbutaline, pirbuterol, procaterol, metaproterenol, fenoterol, bitolterol mesylate, ritodrine, isoprenaline, (long-acting) salmeterol, formoterol, bambuterol, clenbuterol, (ultra-long-acting) indacaterol (asthma, other effects: vasodilation in muscle and liver, relaxation of uterine muscle, and release of insulin) Antagonists: butoxamine, timolol, propranolol (glaucoma, heart attacks, hypertension, migraine headache; investigational: stage fright, post-traumatic stress disorder)
β3			Adipose tissue, gall bladder, small intestine, stomach, prostate, left atrium, bladder, endothelium of coronary micro arteries	Lymphocytes, macrophages, neutrophils	Agonists: amibegron (investigational: antidepressant, anxiolytic), solabegron (overactive bladder, iritable bowel syndrome) Antagonists: SR 59230A

ARs, adrenergic receptors; NK, natural killer.

Each AR subtype couples to a distinct heterotrimeric G protein, triggering conserved downstream signaling cascades that drive core functional effects in innate immune regulation. Coupling of α1-ARs to Gq/11 proteins is the predominant mechanism, which results in activation of phospholipase C (PLC) and subsequent elevation of intracellular calcium levels. This increase, in turn, mediates inflammatory modulation and barrier function regulation (17, 41). In contrast, coupling of α2-ARs to Gi proteins predominantly drives inhibition of adenylate cyclase, thereby reducing production of cyclic adenosine monophosphate (cAMP) (17, 36, 41). By contrast, coupling of β-ARs to Gs proteins acts as the primary stimulus for adenylate cyclase activity, leading to intracellular cAMP accumulation. This, in turn, activates protein kinase A (PKA) and its downstream effector cAMP response element-binding protein (CREB), which regulate transcription of genes involved in inflammatory responses and cell survival (19, 36, 41, 42). In addition to the well-established canonical cAMP-PKA axis, β-ARs signal via non-canonical pathways: G protein-coupled receptor kinase (GRK)-mediated phosphorylation of the β-AR C-terminus generates docking sites for β-arrestin, which both uncouples the receptor from G proteins to drive desensitization and activates alternative downstream signaling cascades, including the mitogen-activated protein kinase (MAPK) pathway that mediates non-canonical immune regulation (36, 43–45). Notably, non-canonical β2-AR–β-arrestin-2 signaling exerts dominant anti-inflammatory effects in neutrophils via direct inhibition of nuclear factor kappa-B (NF-κB) nuclear translocation, and represents the primary mechanism underlying central neural regulation of neutrophil function during ALI (24). The final common endpoint of these signaling cascades is the regulation of inflammatory gene expression, encompassing pro-inflammatory cytokines (TNF-α, IL-1β, IL-6), anti-inflammatory cytokines (IL-10), and chemokines—all of which are core mediators of ALI/ARDS progression (41). This paper summarizes the extant research on ARs agonists and antagonists for bacterial pneumonia and bacterial sepsis-ALI/ARDS (15, 20, 46–53) (**Table 2**).

**Table 2:** The agonists and antagonists of adrenergic receptors used in the treatment of bacterial pneumonia and bacterial sepsis-associated ALI/ARDS.

ARs	Subtypes	Drugs	Effects on ARs	Induction method of models	Regulation of innate immune response-related cells	Protective effects against ALI/ARDS				Relevant signaling molecules/pathways	Ref.
						Tissue Injury	Alveolar Capillary Barrier	Inflammatory response	Physiological dysfunction		
α1-ARs	Not listed	Phenylephrine	Activated	C57BL/6J mice, LPS (2mg/kg) i.t.	Macrophage *in vitro*: TNF-α↓, IL-6↓, CXCL2/MIP-2↓, IL-10↑	Lung injury scores↓	BALF: albumin↓	BALF: counts of total inflammatory cells↓, counts of neutrophils↓, TNF-α↓, IL-6↓, CXCL2/MIP-2↓	Not listed	Macrophage *in vivo*: p-p65/p65↓, p-ERK/ERK↑	(20)
α2-ARs	Not listed	UK14304	Activated	Long–Evans rats, IgG IC-induced alveolitis, LPS (300 μg) i.t.	Not listed	Lung injury↑	Not listed	Not listed.	Not listed	NE from phagocyte	(46)
	Not listed	RX821002	Inhibited	Long–Evans rats, IgG IC-induced alveolitis, LPS (300 μg) i.t.	Macrophage *in vitro*: TNF-α↓, IL-6↓, IL-1β↓, CINC-1↓	Lung injury↓	Not listed	Not listed.	Not listed	NE from phagocyte	(46)
	Not listed	Yohimbine	Inhibited	Sprague-Dawley rats, LPS (300 μg) i.t.	Not listed	Lung injury scores↓	Not listed	BALF: count of white blood cells↓, TNF-α↓, IL-1β↓, IL-6↓	PaO_2_/FiO_2_↑	BALF of ALI rats: NE↑	(47)
	Not listed	Dexmedetomidine	Activated	C57BL/6 mice, LPS (5mg/kg) i.p.	Macrophage *in vitro*: TNF-α↓, IL-1β↓	Lung injury scores↓	Lung W/D↓	Serum: TNF-α↓, IL-1β↓	Not listed	Lung: NLRP3↓, Cleaved Caspase‐1↓	(51)
	Not listed	Dexmedetomidine	Activated	Sprague-Dawley rats, LPS (5mg/kg) i.t.	Not listed	Lung injury scores↓ Lung: SOD↑, CAT↑, MDA↓ Serum: SOD↑, CAT↑, MDA↓	Not listed	Serum: TNF-α↓, IL-1β↓, IL-6↓, IL-5↓, IL-18↓, IL-17↓	Not listed	Lung: Sirt3↑, p-LKB1/LKB1↑,p-AMPK/AMPK↑	(53)
	α2A-ARs	BRL-44408	Inhibited	Sprague-Dawley rats, CLP	Not listed	Lung injury scores↓ MPO↓	Lung W/D↓	Serum: TNF-α↓, IL-1β↓, IL-6↓ Lung: TNF-α↓, IL-6↓	Not listed	Serum of ALI rats: NE↑ Lung: HMGB-1↓, NF-κB↓	(48)
	α2A-ARs	BRL-44408	Inhibited	C57BL/6 mice, LPS (5mg/kg) i.t.	Not listed	Lung injury scores↓	Lung W/D↓ Lung: total protein content↓	BALF: TNF-α↓, IL-6↓	Not listed	BALF of ALI mice: NE↑ Lung: p-MEK↓, p-ERK↓	(50)
	α2A-ARs	*Adra2a^-/-^* mice (*in vivo*), BRL-44408 (*in vitro*)	Inhibited	C57BL/6J mice, LPS (0.5mg/kg) i.t.	Macrophage *in vitro*: TNF-α↓, IL-6↓, IL-10↓	Lung injury scores↓	Lung W/D↓ BALF: albumin↓	BALF: counts of total inflammatory cells↓, counts of neutrophils↓, TNF-α↓, IL-6↓, CXCL2/MIP-2↓	Not listed	BALF of ALI *Adra2a^-/-^* mice: NE↑ Lung: p-p65/p65↓	(52)
β-ARs	β1-ARs, β2-ARs	Propranolol	Inhibited	Mice, 30 μL *Escherichia coli* (10^7^ colony-forming units) i.t.	Not listed	Lung injury↑	Lung W/D↑	Plasma: MIP-2↑	Arterial blood gases: pH↓, PaO_2_↓, PaCO_2_↑	Plasma of ALI mice: EPI↑, NE↑	(15)
	β2-ARs	ICI 118,551	Inhibited	C57BL/6 mice, LPS (10mg/kg or 20mg/kg) i.p., and (60μg in 30 μL) i.t.	Macrophage *in vitro*: iNOS↑, arginase-1↓	Survival↓	BALF: albumin↑	BALF: neutrophil numbers↑, TNF-α↑	Not listed	EPI could protect ALI mice	(49)

AMPK, adenosine monophosphate-activated protein kinase; ARs, adrenergic receptors; BALF, bronchoalveolar lavage fluid; CAT, catalase; CINC-1, cytokine-induced neutrophil chemoattractant 1; CLP, cecal ligation and puncture; CXCL, CXC chemokine ligand; EPI, epinephrine; ERK, extracellular signal-regulated kinases; HMGB-1, high mobility group box 1; IL, interleukin; iNOS, inducible nitric oxide synthase; LKB1, liver kinase B1; LPS, lipopolysaccharide; MDA, malondialdehyde; MEK, mitogen-activated protein kinase kinase; MIP-2, macrophage inflammatory protein 2; MPO, myeloperoxidase; NE, norepinephrine; NF-κB, nuclear factor kappa-B; NLRP3, NOD-like receptor family pyrin domain containing 3; SOD, superoxide dismutase; TNF-α, tumor necrosis factor-alpha; W/D, Wet/Dry weight ratio.

A longstanding challenge in the field is the context-dependent bidirectional effect of AR signaling in ALI/ARDS, termed the “adrenergic paradox.” While AR activation can suppress excessive inflammatory cascades and preserve barrier integrity during early disease stages, it may also impair bacterial clearance, suppress phagocytic function, induce receptor desensitization, and elevate secondary infection risk in advanced disease stages. The anti-inflammatory and tissue-protective effects of AR signaling, particularly those mediated by β2-AR activation, are well characterized in preclinical models. Specifically, β2-AR activation has been shown to suppress pro-inflammatory cytokine production (including TNF-α and IL-1β), while augmenting secretion of the anti-inflammatory cytokine IL-10, via inhibition of NF-κB-driven inflammatory pathways (54, 55). AR signaling also preserves alveolar epithelial and vascular endothelial barrier integrity, enhances alveolar fluid clearance to reduce pulmonary edema, promotes macrophage polarization toward an anti-inflammatory M2 phenotype to drive inflammation resolution, and inhibits excessive neutrophil recruitment to mitigate bystander tissue damage (49, 56–66). However, accumulating evidence demonstrates that AR signaling also exerts profound detrimental effects that must be critically considered in therapeutic development. β2-AR agonists markedly inhibit the bactericidal activity of alveolar macrophages (AMs) and neutrophils, thereby impairing the host’s capacity to clear the primary bacterial infection driving ALI/ARDS (67, 68). β2-AR activation also drives the generation of “trained but paralyzed” AMs, which suppress excessive inflammation yet persist for weeks post-infection and elevate severe secondary pneumonia risk (22, 69–71). Notably, other studies have also demonstrated that β2-AR agonists can potentiate pro-inflammatory cytokine (IL-6, CXCL8) release from neutrophils, and prolong neutrophil lifespan via apoptosis inhibition—effects that may exacerbate inflammation during specific disease stages (72–74).

Recent research has resolved this paradox at both the cellular and molecular levels, demonstrating that divergent functional outcomes of AR signaling within the same immune lineage arise from three core determinants: cellular subset identity, spatial niche, and downstream signaling preference. For macrophages, resident AMs respond to β2-AR activation with anti-inflammatory polarization (49, 65, 75), whereas monocyte-derived IM subsets adjacent to sympathetic nerves undergo pro-inflammatory activation via MAPK signaling following β2-AR engagement (21). For neutrophils, acute physiological NE signaling drives anti-inflammatory responses via β2-AR–β-arrestin-2-mediated inhibition of NF-κB (24), whereas β2-AR agonists can potentiate the release of pro-inflammatory cytokines including IL-6 and CXCL8 from neutrophils, and prolong neutrophil lifespan via inhibition of apoptosis (72–74). This subset- and context-dependent signaling wiring explains the opposing phenotypes observed across studies. Conflicting findings in the field are therefore driven by receptor subtype specificity, disease stage and intervention timing, and experimental variables including model systems, drug administration routes, and dosing regimens.

## 4 Cell-type-specific and context-dependent regulation of pulmonary innate immunity by adrenergic receptors

The functional outcomes of AR signaling in ALI/ARDS are highly cell-type-specific, with distinct effects in non-professional immune cells that form the pulmonary barrier and professional innate immune cells that mediate host defense and inflammation.

### 4.1 Bronchial epithelial cells

Within the pulmonary epithelial compartment—the primary site of injury in pneumonia-induced direct ALI/ARDS—bronchial epithelial cells (BECs) line the airway lumen and constitute the first physical barrier against inhaled pathogens, playing a pivotal role in maintaining airway homeostasis and initiating innate immune responses (76, 77). BECs establish a tight barrier via intercellular junctions, mediate mucociliary clearance through ciliated and mucous cells to capture and eliminate inhaled pathogens, and rapidly recognize microbial PAMPs via PRRs including TLR1–TLR6 and TLR9, triggering the secretion of antimicrobial peptides such as defensins and LL-37, alongside inflammatory cytokines that recruit leukocytes to the site of infection (22, 78–90). BECs express functional β2-ARs, the predominant AR subtype governing BECs innate immune function: β2-AR agonists, including formoterol, salmeterol, and albuterol, suppress cytokine secretion induced by TLR2 (zymosan) and TLR4 (LPS) activation in BECs (91, 92). Exchange protein directly activated by cAMP 1 (Epac1), a key downstream effector of β-AR signaling, also inhibits TLR4/HMGB1-mediated NOD-like receptor family pyrin domain containing 3 (NLRP3) inflammasome activation in BECs infected with *Haemophilus influenzae*, thereby limiting inflammatory amplification (42, 93); however, *Haemophilus influenzae* infection has been shown to downregulate β-AR expression on BECs by 36%, blunting cellular responsiveness to β-AR agonists (94).

### 4.2 Alveolar epithelial cells

Deep within the lung parenchyma, AECs—including type I (AECI) and type II (AECII) cells—form the alveolar epithelial barrier responsible for mediating gas exchange, while also serving as critical regulators of pulmonary innate immunity via both antimicrobial responses and immune tolerance mechanisms (95, 96). AECs predominantly express β2-ARs, which are the master regulators of AECs function in ALI/ARDS (97–99). TLR2 and TLR4 have been identified as expressed in AECs (100). Activation of β2-ARs inhibits TLR2 and TLR4 signaling, thereby suppressing the secretion of pro-inflammatory cytokines including TNF-α (92, 101–103); the non-selective β-AR agonist isoproterenol upregulates β2-AR expression to attenuate LPS-induced TNF-α release, whereas the selective β2-AR agonist fenoterol modulates CD14/TLR4 complex trafficking via β-arrestin 2 to suppress inflammatory signaling (102, 103). In addition to these anti-inflammatory effects, activation of β-ARs modulates multiple programmed cell death modalities in AECs, including pyroptosis, apoptosis, and PANoptosis, through inhibition of inflammatory signaling; this in turn potently suppresses AEC death, promotes AECII-mediated regeneration of the alveolar epithelial barrier, and attenuates alveolar-capillary barrier disruption, effects that are tightly linked to improved ALI/ARDS prognosis (64, 104–111). β2-AR activation further augments alveolar fluid clearance via upregulation of epithelial sodium channel (ENaC) and Na^+^/K^+^-ATPase activity through MAPK/extracellular signal-regulated kinase (ERK) and rapamycin-sensitive signaling pathways, thereby reducing alveolar edema, a pathological hallmark of ALI/ARDS (57, 58, 61, 112–115), and drives pulmonary surfactant secretion from AECII via cAMP-dependent pathways; these surfactant proteins (SPs) lower alveolar surface tension, exert direct bactericidal activity, and modulate alveolar macrophage function (69, 116, 117).

### 4.3 Vascular endothelial cells

In the setting of sepsis-induced indirect ALI/ARDS, the primary cellular target of AR-mediated regulation is the pulmonary vascular endothelium, which lines the luminal surface of the pulmonary vasculature and constitutes the critical barrier limiting vascular leakage and alveolar edema (118, 119). Pulmonary vascular ECs predominantly express β2-ARs, the master regulators of endothelial function in the context of sepsis (120). LPS stimulation downregulates β2-AR expression and upregulates TLR4 expression in human pulmonary microvascular ECs (HPMECs), whereas the β-AR agonist Compound 49b inhibits TLR4 and its downstream MyD88–IL-1 receptor-associated kinase 1 (IRAK1)–tumor necrosis factor receptor-associated factor 6 (TRAF6)–NF-κB signaling cascade, thereby attenuating pro-inflammatory cytokine and adhesion molecule expression in ECs (120, 121). β2-AR activation further attenuates microvascular hyperpermeability and alveolar edema (63), and promotes endothelial repair via enhancement of endothelial progenitor cell function (122). Notably, mice with *Adrb2* (the gene encoding β2-ARs) knockout exhibit increased mortality following sublethal LPS challenge, accompanied by elevated serum TNF-α and diminished IL-10 levels; this phenotype is fully rescued by exogenous IL-10 administration (55), highlighting the non-redundant protective role of endothelial β2-AR signaling in sepsis-induced ALI.

### 4.4 Macrophages

Beyond non-professional immune cells, ARs serve as key regulators of professional innate immune cells in the lung. AMs are the most abundant immune cell population in the pulmonary compartment under homeostatic conditions, and act as central orchestrators of both inflammatory initiation and resolution (22). AMs are broadly categorized into two populations: long-lived resident AMs, which maintain a quiescent, anti-inflammatory M2 phenotype under homeostatic conditions, and recruited monocyte-derived AMs, which differentiate into a pro-inflammatory M1 phenotype in response to infection and tissue injury (123–131). AMs express all major AR subtypes (α1, α2, β1, β2), among which β2-ARs are the most extensively characterized (132–134). β2-AR activation in AMs drives polarization toward the anti-inflammatory M2 phenotype via the phosphatidylinositol 3-kinase (PI3K) pathway and Gs signaling, upregulating anti-inflammatory IL-10 secretion and inhibiting M1-related pro-inflammatory cytokine (TNF-α, IL-1β) production through suppression of the ERK1/2 and p38 MAPK pathways (49, 65, 75), while β2-AR inhibition markedly enhances NF-κB activation and pro-inflammatory mediator secretion in AMs (54). α1-AR activation also suppresses excessive inflammatory responses in AMs via inhibition of NF-κB and activation of ERK1/2, thereby mitigating LPS-induced lung injury (20), while the α2-AR agonist dexmedetomidine induces M2 polarization of AMs to attenuate pulmonary inflammation (66). However, consistent with the aforementioned adrenergic paradox, β2-AR agonists including formoterol and salbutamol markedly inhibit the bactericidal activity of AMs via suppression of TNF-α secretion—a process critical for efficient bacterial clearance (67, 68), and prolonged β2-AR activation induces AMs immune paralysis via upregulation of signal regulatory protein α (SIRPα), thereby elevating the risk of secondary bacterial pneumonia (22, 70, 71, 135–137).

In addition to AMs, IMs represent a distinct cell subset whose AR responsiveness is diametrically opposed to that of AMs. A pro-inflammatory IM subset characterized by high β2-AR expression accumulates in close proximity to pulmonary sympathetic nerves during severe pneumonia, and is derived predominantly from recruited monocytes (21). Unlike AMs, these IMs undergo robust pro-inflammatory activation upon β2-AR ligation, secreting high levels of IL-1β, IL-6, TNF-α, C-C motif chemokine ligand 2 (CCL2), and CXCL2—mediators that amplify leukocyte recruitment and the cytokine storm (21). Genetic or pharmacological disruption of β2-AR signaling in this subset markedly attenuates lung injury and improves overall survival (21). This subset-specific dichotomy is driven by three core determinants: developmental origin, spatial proximity to sympathetic nerves, and preferential coupling to pro-inflammatory MAPK signaling (21).

### 4.5 Dendritic cells

Dendritic cells (DCs), the critical antigen-presenting cells that bridge innate and adaptive immunity in the pulmonary compartment, also express multiple AR subtypes, including β2-ARs and α2-ARs (138, 139). Pulmonary DCs are comprised of three primary subsets: conventional DCs (cDCs), which mediate naive T cell activation; plasmacytoid DCs (pDCs), which produce type I interferons; and monocyte-derived DCs (moDCs), which mediate antimicrobial responses (140, 141). NE modulates NF-κB and activator protein 1 (AP-1) signaling in DCs via β2-ARs, reducing IL-12 production and augmenting IL-10 secretion, thereby inhibiting T helper 1 (Th1) cell activation and attenuating inflammatory responses (138, 139, 142), whereas β2-AR activation enhances LPS-induced IL-33 secretion in DCs via the cAMP-PKA pathway, promoting Th2 cell differentiation and anti-inflammatory responses (143). β2-AR signaling further mediates functional crosstalk between nucleotide-binding oligomerization domain 2 (NOD2) and TLR2 in DCs, thereby regulating extracellular bacterial defense (144).

### 4.6 Innate lymphoid cells

ILCs, the critical mucosal innate immune cells that mediate early host defense and inflammatory regulation in the pulmonary compartment, also express ARs—predominantly β2-ARs—which regulate their differentiation and effector function (145–148). ILCs are broadly categorized into cytotoxic natural killer (NK) cells and helper-like ILCs (ILC1, ILC2, ILC3) (147). NK cells, the predominant cytotoxic ILCs in the lung, produce interferon-γ (IFN-γ) and IL-22 to constrain bacterial infection and activate macrophages (149–154), and express β2-ARs, α1-ARs, and α2-ARs that regulate their cytotoxic and cytokine-secreting functions (145, 155). β2-AR signaling enhances NK cell cytolytic and effector functions in the lung, wherein *Adrb2* upregulation downstream of IL-12/signal transducer and activator of transcription 4 (STAT4) signaling is critical for NK cell maturation and function (156, 157), whereas NK cell-specific β2-AR knockout impairs their capacity to modulate macrophage inflammatory cytokine secretion (158); notably, conflicting studies have demonstrated that β2-AR activation can suppress NK cell activity in specific contexts (159).

Helper-like ILC subsets also exert critical, AR-regulated functions in ALI/ARDS: ILC1s produce IFN-γ to mediate antibacterial host defense, with *Haemophilus influenzae* infection driving ILC1-like cell expansion in the lung to amplify inflammatory responses (160–162); ILC2s secrete type 2 cytokines including IL-4, IL-5, and IL-13 to mediate inflammatory response (162), whereas β2-AR deficiency impairs ILC2 responses and β2-AR agonist administration suppresses ILC2 function to attenuate systemic inflammation (148); and ILC3s secrete IL-17A and TNF-α to enhance macrophage bacterial phagocytosis and clear *Klebsiella pneumoniae* infection—a process critical for preventing pneumonia-induced ALI (163–165)—with β2-AR signaling regulating IL-17A production via cAMP-dependent pathways (166, 167).

### 4.7 Neutrophils

Neutrophils, the first responders to bacterial infection whose influx is a hallmark of antibacterial innate immunity, also express functional β2-ARs that regulate their chemotaxis, activation, and survival (168). Excessive neutrophil recruitment and activation are key drivers of ALI progression, and β2-AR agonists—including formoterol, salmeterol, salbutamol, and fenoterol—reduce LPS-induced neutrophil recruitment to the lung and lower neutrophil counts in bronchoalveolar lavage fluid (BALF) via upregulation of intracellular cAMP (56, 59, 60, 62, 169, 170); however, conflicting studies have demonstrated that β2-AR agonists can potentiate pro-inflammatory cytokine release from neutrophils and prolong neutrophil lifespan via inhibition of apoptosis, effects that may amplify inflammation in specific contexts (72–74). Central neural circuits exert direct control over neutrophil behavior during ALI via sympathetically released NE. Activation of hypothalamic CRH neurons enhances pulmonary sympathetic outflow, and NE engages β2-AR on neutrophils to recruit β-arrestin2, which directly inhibits NF-κB, thereby suppressing neutrophil infiltration, phagocytosis, reactive oxygen species (ROS) production, and neutrophil extracellular traps (NETs) formation. Chemical sympathectomy, β2-AR blockade, or β-arrestin2 ablation completely abolishes this neurogenic protection. This pathway accounts for the acute anti-inflammatory role of adrenergic signaling in neutrophils, whereas chronic stimulation drives receptor desensitization and a pro-inflammatory phenotypic bias.

### 4.8 Cell-cell interactions

Beyond cell-intrinsic regulatory effects within discrete cellular populations, AR signaling exerts integrated immunomodulatory functions in ALI/ARDS, orchestrating bidirectional crosstalk between non-professional immune cells and innate immune cells, as well as between distinct innate immune subsets, to shape the initiation, amplification, and resolution of pulmonary inflammation.

BECs, the primary physical and immune barrier against inhaled pathogens in pneumonia-induced direct ALI/ARDS, form a frontline regulatory network with macrophages via dynamic reciprocal interactions. BECs recognize microbial PAMPs via PRRs, secrete inflammatory cytokines and chemokines to recruit monocyte-derived macrophages, and modulate resident AMs activation state (22, 78–90). β2-ARs, the predominant AR subtype governing BEC innate immune function, serve as the core upstream regulator of this crosstalk: β2-AR agonists suppress TLR2- and TLR4-induced cytokine and chemokine secretion in BECs, thereby indirectly inhibiting macrophage recruitment and excessive pro-inflammatory activation (91, 92). The β-AR downstream effector Epac1 also inhibits TLR4/HMGB1-mediated NLRP3 inflammasome activation in BECs during *Haemophilus influenzae* infection, limiting inflammatory amplification and pro-inflammatory macrophage polarization (42, 93), whereas *Haemophilus influenzae* infection downregulates BEC β-AR expression by 36% to blunt this inhibitory effect (94).

Deep within the lung parenchyma, AECs and AMs constitute the core cellular network maintaining alveolar homeostasis, with reciprocal interactions throughout ALI/ARDS progression. AECs secrete cytokines and chemokines to recruit and modulate AM function, whereas AMs in turn regulate epithelial barrier repair and alveolar inflammation (171–173). AR signaling serves as a critical upstream regulator of this axis: β2-AR activation suppresses TLR2/TLR4-mediated pro-inflammatory cytokine and chemokine secretion in AECs, attenuating AM recruitment and M1 polarization (55, 92, 102, 103); SPs from AECII, whose production is tightly regulated by β2-AR/cAMP signaling, further enhance AM bactericidal activity and fine-tune alveolar inflammation (69, 116).

In sepsis-induced indirect ALI/ARDS, pulmonary vascular ECs form another key regulatory axis with macrophages via AR signaling. Pulmonary vascular ECs predominantly express β2-ARs, the master regulators of endothelial function in sepsis (120). The β-AR agonist Compound 49b inhibits TLR4 and its downstream MyD88–IRAK1–TRAF6–NF-κB cascade, attenuating pro-inflammatory cytokine and adhesion molecule expression in ECs (120, 121), thereby inhibiting circulating monocyte adhesion, transendothelial migration, and macrophage differentiation to limit excessive pulmonary macrophage accumulation and activation. β2-AR activation also attenuates microvascular hyperpermeability, promotes endothelial repair, and indirectly modulates the pulmonary microenvironment to fine-tune macrophage activation (63, 122). Notably, Adrb2 knockout mice show increased mortality following sublethal LPS challenge, with elevated serum TNF-α and reduced IL-10, a phenotype fully rescued by exogenous IL-10 administration (55), highlighting the non-redundant role of endothelial β2-AR signaling in macrophage-associated inflammatory networks.

Beyond structural cell-macrophage crosstalk, AMs and neutrophils form the core inflammatory amplification network in ALI/ARDS, which is tightly modulated by AR signaling. AMs secrete chemokines including CXCL2 and IL-8 to recruit neutrophils to inflammatory foci, whereas neutrophil-derived mediators drive AM M1 polarization, forming a pathological positive inflammatory feedback loop (174–176). AR signaling disrupts this loop via multiple non-redundant mechanisms: β2-AR activation inhibits AM secretion of neutrophil-targeted chemokines to reduce neutrophil recruitment (54); α1-AR activation suppresses AM NF-κB signaling, and α2-AR activation promotes AM M2 polarization, both of which dampen inflammatory amplification between the two cell types (20, 66).

### 4.9 Integrated adrenergic immunoregulatory networks in ALI/ARDS

**Figure 2:**
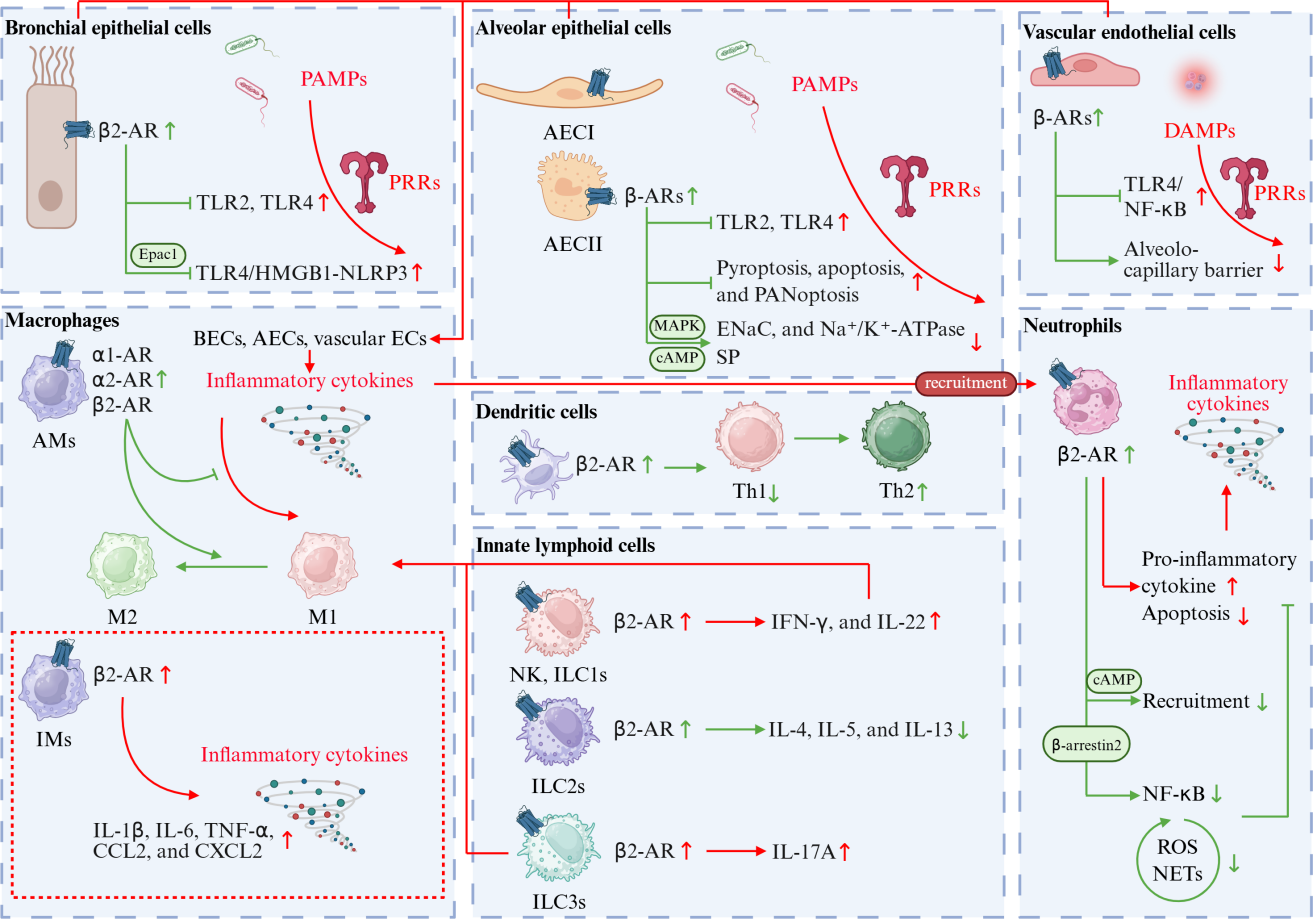
The regulatory role of adrenergic receptors in innate immune responses of pulmonary cells. AECs, alveolar epithelial cells; AMs, alveolar macrophages; AR, adrenergic receptor; BECs, bronchial epithelial cells; CCL, C-C motif chemokine ligand; CXCL, CXC chemokine ligand; DAMPs, damage-associated molecular patterns; ECs, endothelial cells; ENaC, epithelial sodium channel; Epac1, exchange protein directly activated by cAMP 1; HMGB-1, high mobility group box 1; IFN-γ, interferon-γ; IL, interleukin; ILCs, innate lymphoid cells; IMs, interstitial macrophages; NETs, neutrophil extracellular traps; NF-κB, nuclear factor kappa-B; NK, natural killer; NLRP3, NOD-like receptor family pyrin domain containing 3; PAMPs, pathogen-associated molecular patterns; PRRs, pattern recognition receptors; ROS, reactive oxygen species; SP, surfactant protein; Th1, T helper 1 cell; Th2, T helper 2 cell; TLR, Toll-like receptor; TNF-α, tumor necrosis factor-alpha.

ARs exert cell-type-specific, context-dependent regulatory control over pulmonary innate immunity in ALI/ARDS, with divergent functional outcomes across non-professional immune cells and professional innate immune subsets (**Figure 2**). β2-AR is the predominant functional subtype mediating these effects: in BECs and AECs, the primary injury targets in pneumonia-induced direct ALI/ARDS, β2-AR activation suppresses TLR2/4-driven inflammatory signaling and NLRP3 inflammasome activation, while concurrently preserving alveolar barrier integrity, promoting epithelial regeneration, enhancing alveolar fluid clearance, and driving surfactant secretion. In pulmonary vascular ECs, the key regulatory target in sepsis-induced indirect ALI/ARDS, β2-AR signaling inhibits the TLR4-MyD88-NF-κB cascade, attenuates microvascular hyperpermeability, and confers non-redundant survival protection against endotoxemic lung injury. Within professional innate immune compartments, AR signaling drives subset-specific bidirectional effects: β2-AR activation in AMs promotes anti-inflammatory M2 polarization, yet prolonged stimulation triggers immune paralysis and impairs bactericidal capacity; by contrast, a high β2-AR-expressing IMs subset undergoes robust pro-inflammatory activation upon receptor ligation to amplify pulmonary inflammation. AR signaling also modulates the functional phenotype of DCs, ILCs subsets, and neutrophils, with context-dependent pro- or anti-inflammatory effects dictated by stimulation duration, cellular developmental origin, and local tissue microenvironment. Beyond cell-intrinsic actions, AR signaling orchestrates bidirectional crosstalk between non-professional immune cells and innate immune populations, as well as between discrete innate immune subsets, to shape the initiation, amplification, and resolution of ALI/ARDS-associated inflammation. Collectively, this cell- and context-specific regulatory framework resolves the well-documented adrenergic paradox in ALI/ARDS, establishing a mechanistic blueprint for the rational development of cell-targeted adrenergic therapeutics for this high-mortality syndrome.

## 5 Translational implications of targeting adrenergic receptors in ALI/ARDS: challenges and opportunities

Despite extensive preclinical evidence validating the protective effects of ARs-targeted therapies in ALI/ARDS, clinical translation of these strategies has been largely unsuccessful, representing a critical barrier to clinical implementation of this pathway for patient care. The most extensively characterized ARs-targeted agents in ARDS are β2-AR agonists, which exhibit robust protective efficacy in preclinical disease models, yet have failed to demonstrate definitive clinical benefit in large-scale randomized controlled trials (RCTs). The landmark BALTI-2 trial—a multicenter, randomized, double-blind, placebo-controlled trial evaluating intravenous salbutamol (a selective β2-AR agonist) in mechanically ventilated patients with early ARDS—was terminated prematurely due to significantly higher 28-day mortality in the salbutamol cohort, coupled with reduced ventilator-free and organ failure-free days, as well as an increased incidence of cardiovascular and metabolic adverse events. These findings demonstrate that systemic intravenous administration of β2-AR agonist provides no clinical benefit and may exert harmful effects in patients with ARDS, directly contraindicating its routine clinical use (177). Notably, several ARs-targeted clinical trials for ALI/ARDS are currently active in recruitment or in preparatory phases, including trials evaluating β2-AR agonists (NCT05527704), α2A-AR and α2B-AR agonists (NCT05241067), and β1-AR blockers (NCT06013319, NCT05847517). These trials are rationally designed to address key limitations of prior studies via optimized dosing regimens, prospectively defined patient stratification, and alternative administration routes.

### 5.1 Core barriers to clinical translation

Four interrelated, mechanism-driven challenges have undermined the clinical development of ARs-targeted therapies for ARDS, all of which stem from a fundamental failure to integrate context-dependent AR biology into clinical trial design.

First, route-dependent mismatch between drug exposure and the therapeutic target tissue represents the most immediate barrier. Preclinical studies have predominantly utilized lung-localized delivery to achieve high parenchymal drug concentrations with minimal systemic exposure, whereas failed phase III trials employed continuous intravenous infusion of β2-AR agonists (177). Systemic administration results in negligible lung bioavailability, necessitating high doses that drive dose-limiting cardiovascular toxicity in the heart. Even more critically, systemic delivery triggers non-selective AR activation across all pulmonary cell types, simultaneously engaging protective signaling in alveolar epithelial cells, ECs, and neutrophils, and pro-inflammatory signaling in the monocyte-derived interstitial macrophage subset that expands during severe pneumonia. This non-specific activation directly abrogates therapeutic benefit and can drive net pathological inflammation.

Second, misalignment of intervention timing with the therapeutic window fundamentally limits therapeutic efficacy. Preclinical studies almost universally employ prophylactic or early intervention during the initiation phase of inflammatory injury, when AR signaling exerts its most potent barrier-protective and anti-inflammatory effects via the hypothalamic paraventricular nucleus (PVN) CRH neuron-driven sympathetic circuit. In contrast, clinical trials have exclusively enrolled patients with established Berlin-defined ARDS, typically 24–48 hours after the inciting pathological insult. By this disease stage, the inflammatory cascade is fully amplified, irreversible alveolar-vascular barrier damage has occurred, and the pro-inflammatory CeA GABAergic neuron-sympathetic circuit dominates pulmonary AR signaling, rendering β2-AR agonists ineffective or even harmful.

Third, profound patient heterogeneity and the absence of precision patient stratification have diluted therapeutic signals. ARDS is a heterogeneous syndrome with two etiologically and immunologically distinct subtypes: direct lung injury (e.g., bacterial pneumonia), characterized by primary epithelial damage and expansion of pro-inflammatory β2-AR-high interstitial macrophages, and indirect lung injury (e.g., non-pulmonary sepsis), driven by systemic inflammation and primary endothelial injury. Prior clinical trials enrolled unselected, all-comer patient cohorts. Additional heterogeneity arising from age, comorbidities (which drive chronic sympathetic activation and AR desensitization), and inflammatory phenotypes (hyperinflammatory vs hypoinflammatory) further confounded trial outcomes, masking potential therapeutic benefits in treatment-responsive subgroups.

Fourth, underappreciated receptor desensitization, biased signaling, and the aforementioned adrenergic paradox have undermined both therapeutic efficacy and safety. As GPCRs, β2-AR undergo rapid desensitization, internalization, and degradation following continuous agonist exposure—an inherent limitation of the continuous infusion regimens used in all failed clinical trials. Sustained agonist exposure also shifts β-arrestin function from anti-inflammatory signal transduction (via NF-κB inhibition in neutrophils) to receptor desensitization, thereby ablating the primary protective arm of β2-AR signaling. Most critically, prior trials entirely ignored the core tenet of the adrenergic paradox: non-selective β2-AR activation simultaneously suppresses excessive inflammation and impairs the phagocytic and bactericidal capacity of AMs and neutrophils. In patients with active bacterial infection, this immunosuppressive effect results in impaired pathogen clearance, increased bacterial burden, and elevated mortality.

### 5.2 Rational therapeutic strategies informed by mechanistic advances

Recent insights into AR-mediated regulation of pulmonary innate immunity have delineated four core, mechanism-driven strategies to overcome longstanding translational barriers and unlock the therapeutic potential of ARs targeting in ARDS.

First, lung-localized inhaled delivery represents the most immediate, high-impact strategy for therapeutic optimization. Inhaled administration achieves high, uniform drug concentrations throughout the lung parenchyma with negligible systemic absorption, thereby eliminating the dose-limiting cardiovascular toxicity associated with intravenous delivery.

Second, shifting the therapeutic window to early intervention in at-risk patients is critical to maximize therapeutic efficacy. Rather than targeting patients with established, late-stage ARDS, future clinical trials should enroll patients at high risk of ARDS—identified via validated risk stratification tools such as the Lung Injury Prediction Score—with intervention initiated within 6 hours of the inciting pathological insult. This early intervention engages the protective PVN-driven sympathetic circuit prior to the dominance of the pro-inflammatory CeA pathway, thereby blocking the initiation of the inflammatory cascade and preventing progression to full-blown ARDS.

Third, biomarker-guided precision patient stratification is essential to address the profound heterogeneity of ARDS. Future clinical trials must stratify patients along three core dimensions: (1) ARDS etiology (direct vs indirect lung injury), with separate parallel study cohorts and tailored, subtype-specific interventions; (2) inflammatory phenotype, with enrichment for the hyperinflammatory subgroup most likely to derive benefit from anti-inflammatory AR modulation; and (3) AR pathway activity, using biomarkers including circulating leukocyte β2-AR expression, bronchoalveolar lavage fluid norepinephrine levels, and β-arrestin activity to exclude patients with desensitized or non-responsive AR signaling. This precision medicine approach eliminates non-responsive patient populations and maximizes the statistical power to detect a meaningful therapeutic signal.

Fourth, the development of cell-type-selective and pathway-biased AR ligands will overcome off-target effects and resolve the adrenergic paradox at the molecular level. Next-generation agents, including β-arrestin2-biased β2-AR agonists, can selectively engage anti-inflammatory non-canonical signaling in neutrophils. Complementary strategies include epithelial- or endothelial-specific β2-AR agonists, tailored to pneumonia-induced and sepsis-induced ALI, respectively. For infection-associated ARDS, rational combination therapy that pairs AR modulation with pathogen-directed antibiotics is essential: ARs-targeted agents suppress pathological inflammation, while antibiotics preserve host defense and prevent unchecked bacterial outgrowth. Synergistic co-administration with other mechanistically distinct anti-inflammatory agents further enhances therapeutic efficacy and reduces the required effective dose, thereby minimizing both off-target toxicity and the risk of receptor desensitization.

Despite the disappointing outcomes of prior clinical trials, AR signaling remains one of the most biologically validated and druggable therapeutic targets for ALI/ARDS. The failure of historical clinical studies stemmed from an incomplete mechanistic understanding of context-dependent AR function, rather than a lack of validity of the therapeutic target itself. Recent breakthroughs delineating the neural regulation of pulmonary AR signaling, cell subset-specific bidirectional effects, and biased downstream signaling cascades have provided a clear roadmap for rational therapeutic development. By implementing lung-localized delivery, early intervention, precision patient stratification, and next-generation biased ligands, ARs-targeted therapies hold the potential to finally deliver meaningful clinical benefit for patients with ARDS, addressing a major unmet medical need in critical care medicine.

## 6 Conclusions and future perspectives

Bacterial pneumonia and sepsis are the leading causes of ALI/ARDS, with disease progression driven by dysregulated pulmonary innate immune responses. ARs, which are broadly expressed across all key structural and immune cell populations in the lung, serve as the core interface between the SNS and pulmonary innate immunity, mediating bidirectional, cell-type-specific, and context-dependent effects on ALI/ARDS progression. The adrenergic paradox—defined as the context-dependent balance between the anti-inflammatory and barrier-protective effects of AR signaling and its detrimental impacts on bacterial clearance and immune paralysis—represents the central consideration for both basic mechanistic research and therapeutic development in this field. The regulatory effects of ARs are exquisitely dependent on disease etiology: in pneumonia-induced direct ALI/ARDS, ARs primarily target the alveolar epithelial barrier, whereas in sepsis-induced indirect ALI/ARDS, ARs mainly regulate the pulmonary vascular endothelial barrier, with distinct optimal therapeutic strategies for each etiology. Recent advances in neuroimmunology have positioned AR signaling within a hierarchical central–peripheral regulatory network, revealing that opposing brain–lung sympathetic circuits dictate the balance between protective and pathological inflammation. This mechanistic framework transforms our conceptual understanding of adrenergic regulation in ALI/ARDS and enables the rational design of next-generation ARs-targeted therapies.

The failure of preclinical findings to translate to meaningful clinical benefit is primarily driven by suboptimal drug delivery, misaligned intervention timing, unaccounted patient heterogeneity, and a failure to address the core tenets of the adrenergic paradox—all limitations that can be directly mitigated by the optimized therapeutic strategies outlined herein. Looking forward, several key research directions will advance the field: the application of single-cell sequencing and spatial transcriptomics to map dynamic changes in AR expression across innate immune cell subpopulations throughout the full disease course of ALI/ARDS, to delineate the subset-specific and cell-type-specific functions of ARs; further mechanistic dissection of neuro-immune crosstalk along the brain–lung axis, including the respective roles of the sympathetic and parasympathetic nervous systems in regulating pulmonary AR signaling and innate immune responses, as well as the therapeutic potential of neurostimulation modalities including vagus nerve stimulation (VNS) and electroacupuncture; the development of nanoscale delivery systems for ARs-targeted therapeutics, to achieve cell-type-specific, lung-localized drug delivery, thereby enhancing therapeutic efficacy and minimizing systemic adverse effects; and the extension of this mechanistic framework to non-infectious etiologies of ALI/ARDS, including trauma, aspiration, and mechanical ventilation, to broaden the clinical applicability of ARs-targeted therapies. Future therapeutic development should prioritize pathway-biased ligands that selectively engage protective signaling modules—such as β-arrestin2–biased β2-AR agonists targeting neutrophils—while avoiding pro-inflammatory signaling in interstitial macrophage subsets. These precision medicine strategies hold the potential to finally unlock the clinical promise of adrenergic modulation for patients with ALI/ARDS.

In summary, ARs represent a promising, highly druggable therapeutic target for ALI/ARDS, but successful clinical translation requires a refined, mechanistic understanding of the context-dependent adrenergic paradox, optimized therapeutic strategies, and rigorously designed clinical trials incorporating appropriate patient stratification and precisely timed intervention. With continued mechanistic and translational research in these areas, ARs-targeted therapies hold the potential to finally deliver meaningful clinical benefit to patients with ALI/ARDS, addressing a longstanding, critical unmet need in critical care medicine.

## Glossary

AECs: alveolar epithelial cells
ALI: acute lung injury
AMPK: adenosine monophosphate-activated protein kinase
AMs: alveolar macrophages
AP-1: activator protein 1
ARDS: acute respiratory distress syndrome
ARs: adrenergic receptors
BALF: bronchoalveolar lavage fluid
BECs: bronchial epithelial cells
cAMP: cyclic adenosine 2’,3’-monophosphate
CAP: community-acquired pneumonia
CAT: catalase
CCL: C-C motif chemokine ligand
cDCs: conventional DCs
CeA: central amygdala;
CINC-1: cytokine-induced neutrophil chemoattractant 1
CLP: cecal ligation and puncture
CLRs: C-type lectin receptors
CREB: cAMP response element-binding protein
CRH: corticotropin-releasing hormone
CXCL: CXC chemokine ligand
DAMPs: damage-associated molecular patterns
DCs: dendritic cells
ECs: endothelial cells
ENaC: epithelial sodium channel
Epac1: exchange protein directly activated by cAMP 1
EPI: epinephrine
ERK1/2: extracellular signal-regulated kinases 1 and 2
GABA: γ-aminobutyric acid
GPCRs: G protein-coupled receptors
GRK: G protein-coupled receptor kinase
HAP: hospital-acquired pneumonia
HMGB-1: high mobility group box 1
HPMECs: human pulmonary microvascular endothelial cells
IFN-γ: interferon-γ
IL: interleukin
ILCs: innate lymphoid cells
IMs: interstitial macrophages
iNOS: inducible nitric oxide synthase
LKB1: liver kinase B1
LPS: lipopolysaccharide
MAMPs: microbe-associated molecular patterns
MAPK: mitogen-activated protein kinase
MDA: malondialdehyde
MEK: mitogen-activated protein kinase kinase
MIP-2: macrophage inflammatory protein 2
moDCs: monocyte-derived DCs
MPO: myeloperoxidase
NE: norepinephrine
NETs: neutrophil extracellular traps
NF-κB: nuclear factor kappa-B
NK: natural killer
NLRP3: NOD-like receptor family pyrin domain containing 3
NLRs: NOD-like receptors
NOD2: nucleotide-binding oligomerization domain 2
PAMPs: pathogen-associated molecular patterns
pDCs: plasmacytoid DCs
PI3K: phosphoinositide 3-kinase
PKA: protein kinase A
PLC: phospholipase C
PRRs: pattern recognition receptors
RLRs: RIG-I-like receptors
ROS: reactive oxygen species
SAM: sympathetic–adrenal–medullary
SIRPα: signal-regulated protein alpha
SNS: sympathetic nervous system
SOD: superoxide dismutase
SPs: surfactant proteins
STAT: signal transducers and activators of transcription
Th1: T helper 1 cell
Th2: T helper 2 cell
TLRs: Toll-like receptors
TNF-α: tumor necrosis factor-alpha
TRAF6: tumor necrosis factor receptor-associated factor 6
VNS: vagus nerve stimulation
W/D: Wet/Dry weight ratio
